# Impact of Fermentation on Bacterial and Fungal Microbiome Interactions in *Pla-Ra*, a Traditional Thai Food

**DOI:** 10.1155/ijfo/5530574

**Published:** 2025-12-08

**Authors:** Thatsanapong Pongking, Xiuqiang Chen, Keerapach Tunbenjasiri, Lu Zhang, Ratthaphol Kraiklang, Somchai Pinlaor, Arunnee Sangka, Aroonlug Lulitanond, David Blair, Porntip Pinlaor

**Affiliations:** ^1^ Biomedical Sciences Program Graduate School, Khon Kaen University, Khon Kaen, Thailand, kku.ac.th; ^2^ Microbiome Dynamics, Leibniz Institute for Natural Product Research and Infection Biology-Hans Knöll Institute, Jena, Germany; ^3^ Nutrition for Health Program, Faculty of Public Health, Khon Kaen University, Khon Kaen, Thailand, kku.ac.th; ^4^ Department of Parasitology, Faculty of Medicine, Khon Kaen University, Khon Kaen, Thailand, kku.ac.th; ^5^ Centre for Research and Development of Medical Diagnostic Laboratories, Faculty of Associated Medical Sciences, Khon Kaen University, Khon Kaen, Thailand, kku.ac.th; ^6^ College of Science and Engineering, James Cook University, Townsville, Queensland, Australia, jcu.edu.au

**Keywords:** bacteria–fungi interaction, functional pathways, microbial succession, *pla-ra* fermentation

## Abstract

*Pla-ra*, a traditional Thai fermented fish product, undergoes complex microbial and biochemical transformations that contribute to its unique flavor and aroma. This study investigated the effects of fermentation on the microbiome of *pla-ra*, focusing on bacteria–fungi interactions. Freshwater fish, combined with salt and roasted rice, were sampled after 1 and 6 months of fermentation. Bacterial and fungal communities in these *pla-ra* samples were characterized using next‐generation sequencing of the 16S rRNA (V3–V4 region) and ITS2 regions, respectively. Results demonstrated that initial bacterial contamination levels were within standard limits, while fungal contamination (estimated by culture) exceeded *pla-ra* guidelines. Both pH and salinity increased slightly during fermentation. A decrease in bacterial alpha diversity and an increase in fungal diversity during fermentation were observed. Bacterial genera such as *Candidatus Hydrogenedens*, *Bellilinea*, and various unclassified *Acidobacteria* groups declined, while *Enhydrobacter*, *Dermacoccus*, and *Halanaerobium* increased, indicating adaptation to increased salinity. *Penicillium*, the dominant fungal taxon, has a potential role in flavor development. Importantly, microbial network analysis revealed dynamic interactions, including an inhibitory effect of *Penicillium* on *Dermacoccus* and *Enhydrobacter*, but only early in fermentation. KEGG pathway analysis highlighted upregulation of glycerophospholipid metabolism and downregulation of lipid metabolism. In conclusion, our results demonstrate that *pla-ra* fermentation decreases bacterial and increases fungal diversity, impacting bacteria–fungi interactions and correlations in ways that may influence taste and smell. This information contributes to optimizing traditional fermentation practices and enhancing product quality and safety.

## 1. Introduction

*Pla-ra*, a traditional Thai fermented fish product, is particularly popular in the northeastern [1] region of Thailand and plays a significant role in Southeast Asian cuisine. It has an impact on the national economy, with annual production of 40,000 ton/year and exports valued at approximately 800 million Baht [1]. *Pla-ra* is made by combining freshwater fish, salt, and often roasted rice bran or powder. In some cases, previously fermented *pla-ra* or bacterial starter cultures, such as *Bacillus subtilis* subsp. *subtilis* UD6‐2 and *Virgibacillus halodenitrificans* NCFF‐2, are added to promote fermentation [2]. The mixture is then sealed and fermented for several months. This condiment, characterized by its pungent aroma and distinctive umami flavor, is produced through a complex natural fermentation process involving diverse microbial communities [3–5]. It is often associated with beneficial microorganisms like lactic acid bacteria and *Bacillus* species that contribute to the unique flavors and potential health‐promoting properties of *pla-ra* [6]. However, the precise mechanisms by which fermentation‐driven interactions between bacteria and fungi influence flavor and aroma profiles remain understudied.

Fermented foods, particularly those produced through lactic acid fermentation, have been fundamental to food preservation across cultures [7]. Fermentation can be classified into several categories based on the type of end product: alcohol fermentation, acid fermentation, carbon dioxide (bread) fermentation, and amino acid/peptide fermentation [8]. Many fermented fish products in Asia, including *pla-ra*, are produced through lactic acid fermentation, utilizing salt and carbohydrate sources like rice or millet [9]. Such products are characterized by a strong aroma, salty taste, and a sour flavor. The intensity of these attributes depends on the fermentation duration and the amount of salt used in the process [10].

The microorganisms (bacteria and fungi) present during the fermentation process also play a crucial role in influencing the unique smell and taste of *pla-ra*. For example, members of the bacterial taxon *Lactobacillus* enhance the production of volatile compounds such as aldehydes, alcohols, and esters, which are essential for the characteristic taste and aroma of *pla-ra* [11]. *Tetragenococcus*, *Staphylococcus*, and *Lactobacillus* are common bacterial taxa found in *pla-ra* [5, 12], and the fungi include *Densospora*, *Exophiala*, and *Monascus* [12].

Microorganisms present during fermentation also interact with each other in various ways, leading to dynamic changes in the physical and chemical properties of the end product. For instance, *Debaryomyces hansenii* enhances the growth of *Staphylococcus xylosus* by accumulating branched‐chain esters in bacterial cells, as observed in fermented sausages [13]. Similarly, *Tetragenococcus halophilus* facilitates the growth of *Zygosaccharomyces rouxii* by creating an acidic environment that is essential for microbial succession in soy sauce fermentation [14]. Recent advances in culture‐independent techniques, such as next‐generation sequencing (NGS), have enabled a more comprehensive understanding of the bacterial–fungal interactions present in and influencing these traditional fermented foods [15].

This study investigated the changes in the microbiome (bacteria and fungi) of *pla-ra* during fermentation, specifically examining bacterial–fungal interactions. To achieve this, *pla-ra* samples were collected at 1 and 6 months after fermentation. Microbiome and mycobiome changes were analyzed using NGS, targeting the V3–V4 region of 16S rRNA for bacterial communities and the ITS2 region for fungal communities. Our findings help to elucidate the microbial–mycobiome interactions that provide the final characteristics and properties of *pla-ra*.

## 2. Materials and Methods

### 2.1. Sample Collection

Samples were randomly collected from newly prepared material prior to the start of fermentation at *pla-ra* standard factory in Khon Kaen Province, Thailand. The fermentation process utilized two individual 100‐L containers: Container 1, a large jar, covered with a plastic bag, and Container 2, a bucket, sealed with both a lid and a plastic bag. In both containers, *pla-ra* was prepared using various freshwater fish species, salt, and rice bran. No starter culture was added. Samples were collected at two time points, one from each container 1 and 6 months after the start of fermentation, corresponding to early and mature fermentation stages as commonly practiced in traditional *pla-ra* production. After collection, each sample was stored at −20°C for further analysis. From each container and time point, 500 g of *pla-ra* was individually homogenized and three subsamples were randomly taken (*n* = 3 per container; total *n* = 6 per time point). Each subsample underwent independent DNA extraction and library preparation and was analyzed as a separate observation. These triplicates represent biological subsamples that capture within‐container heterogeneity. No technical replicate libraries were generated. This study involved only *pla-ra* samples obtained from a fermentation facility and required no ethical clearance, as no human or animal subjects were involved.

### 2.2. Determination of Initial Microbial Contamination in Pla‐Ra

The possibility of contamination with undesirable microbes was examined only 1 month after the start of fermentation. A 1‐g sample of *pla-ra* was taken from each container and cultured using conventional methods. The cultivation and enumeration of *Bacillus cereus*, *Clostridium perfringens*, *Escherichia coli*, *Salmonella* spp., *Staphylococcus aureus*, yeast, and molds were performed following the Bacteriological Analytical Manual guidelines [16].

### 2.3. pH and Salinity Measurement

The pH and salinity of *pla-ra* samples at both collection time points were determined using a YSI 556 MPS handheld meter (Serial Number 16K102403, YSI Inc., Ohio, United States). Prior to measurement, the YSI 556 MPS meter was calibrated according to the manufacturer’s instructions. The pH readings were recorded by immersing the probe into the sample, allowing the meter to stabilize before noting the value. Salinity was measured using the meter′s conductivity function, which was converted to salinity using the built‐in conversion algorithm.

### 2.4. Genomic Bacterial and Fungal Extraction and Sequencing

Bacterial and fungal DNA was extracted from 250 mg of homogenized *pla-ra* using the Qiagen PowerFecal DNA kit (QIAGEN GmbH, Hilden, Germany) according to the manufacturer′s instructions. The extracted DNA was stored at −20°C to −80°C. DNA concentration and purity were measured using a NanoDrop 2000 spectrophotometer (Thermo Scientific, United States). DNA integrity was verified on a 1.5% agarose gel prepared with Novel Juice supplied in 6× loading buffer (GeneDireX, Taiwan) and visualized using a BIO‐RAD Universal Hood II Gel Doc Imaging System (Bio‐Rad, United States). The V3–V4 regions of 16s rRNA were amplified using the universal primers for prokaryotes 341F (5 ^′^‐CCTAYGGGRBGCASCAG‐3 ^′^) and 806R (5 ^′^‐GGACTACNNGGGTATCTAAT‐3 ^′^) [17]. The second internal transcribed spacer (ITS2) region was amplified using primers ITS3‐2024F (5 ^′^‐GCATCGATGAAGAACGCAGC‐3 ^′^) and ITS4‐2409R (5 ^′^‐TCCTCCGCTTATTGATATGC‐3 ^′^) [18]. Sequencing was done using an Illumina MiSeq platform with paired‐end reads (2 × 250) (Illumina Inc., California, United States). Before each sequencing run, standard Illumina protocols were followed for sterilization and washing of the machine.

### 2.5. Bioinformatics and Statistical Analysis

Raw reads of 16S rRNA and ITS2 were processed using LotuS2 (v2.23) [19, 20] with default settings. For 16S rRNA data, chimeric sequences were detected and removed using VSEARCH‐UCHIME. The clean tags were clustered into operational taxonomic units (OTUs) sharing at least 97% sequence similarity using UPARSE (v11.0). The representative sequences were aligned against a reference 16S ribosomal DNA (rDNA) with the SILVA SSU database release 138 (https://http://www.arb-silva.de/download/arb-fles/). Taxonomic consistency was assessed at each taxonomic level, with a taxonomic assignment accepted by default if over 90% of reference sequences matched the same taxon. Sequences that could not be assigned to any taxonomic level were discarded. For ITS2 data, chimeric sequences were detected and removed using VSEARCH‐UCHIME. Remaining reads were clustered into OTUs sharing at least 97% similarity using UPARSE (v11.0) [21]. The representative sequences of fungal OTUs were mapped to the UNITE ITS taxonomic reference database (v10.0) [22] using the Lambda taxonomic similarity search algorithm. The ITSx (v1.3) program [23] was used to extract ITS2 sequences and remove non‐ITS OTUs.

All statistical analyses and figure generation for microbiome and mycobiome data were performed using R (v4.3.0). Alpha diversity, including observed and Shannon indices, was estimated using the phyloseq package (v1.44.0). Alpha diversity was visualized using ggboxplot from the ggpubr package (v0.6.0). Statistical differences in alpha diversity were assessed using linear regression, controlling for container effects (model: Alpha~group + container). For beta‐diversity analysis, the Bray–Curtis distance was calculated using the vegan package (v2.6.4), and between‐group differences were evaluated using PERMANOVA. Homogeneity of multivariate dispersion was examined using betadisper prior to interpreting PERMANOVA. PERMANOVA used 999 permutations with permutations constrained by container (strata = container) to account for paired sampling. Alpha‐diversity comparisons (per index) and PERMANOVA on a single prespecified distance were treated as single‐hypothesis tests and are reported as *p* (two‐sided, unadjusted). Prior to the analysis of differential abundance, a prevalence filter was applied to remove OTUs present in only a single sample. Subsequently, DESeq2 (v1.40.2) [24] was used with default settings to perform differential abundance analysis. Multiple testing was controlled using the Benjamini–Hochberg (BH) procedure implemented in DESeq2. Adjusted *p* values (*p*adj) were reported, with *p*adj < 0.1 considered significant.

The DGCA R package (v2.0.0) [25] was utilized to construct a network based on differential correlations among bacteria and fungi in *pla-ra* samples, comparing 1 and 6 months after the start of fermentation. The analysis considered bacteria‐with‐bacteria, fungus‐with‐fungus, and fungus‐with‐bacteria interactions, using an empirical *p* value threshold of ≤ 0.05. Following this, the ddMEGENA function in the DGCA R package was employed to identify coexpressed modules within the constructed network, using a module *p* value threshold of ≤ 0.05. These modules were derived from the differentially correlated microbial pairs. Subsequently, Cytoscape software (v3.10.2) [26] was used to visualize the differential correlation analysis at the level of genus.

Functional abundances were predicted from 16S rRNA data using PICRUSt2 (v2.5.2) [27] with the nearest sequenced taxon index (NSTI) cutoff set at 2.0. The KEGG ortholog (KO) abundance data for each sample were then subjected to differential abundance analysis using the DESeq2 method. Visualizations of the results were created using the ggpicrust2 R package (v1.7.3).

Bacterial genera and KEGG pathways showing significant differences through time were included in a Spearman′s rank correlation analysis. Residual values were used for the correlation, performed with the psych R package (v2.4.3). *p* values were adjusted for multiple tests by applying the BH procedure. Correlations were considered significant at *p* < 0.05, with additional notation for adjusted *p* values < 0.1. The correlation heatmap was constructed in the ggplot2 R package (v3.5.1).

## 3. Results and Discussion

### 3.1. Fermentation Changes pH and Salt Content in Pla‐Ra

The pH of samples after 1 month of fermentation was 6.42 ± 0.27 and 7.06 ± 0.08 after 6 months. At the same time points, the salinity levels were 47.98 ± 3.12 parts per thousand (ppt) and 48.74 ± 1.15 ppt, respectively. Although slight, the changes in pH and salt content indicate alterations in microbial communities and their metabolic activity during *pla-ra* fermentation. At 1 month, bacterial counts were within the Thai Agricultural Standard safety limit for *pla-ra*, while fungal levels exceeded the acceptable guideline (Table 1). High fungal contamination during early fermentation necessitates further investigation and regulation of the mycological composition of this fermented food to ensure consumer safety and food quality [15, 29]. See below for further discussion of this.

**Table 1:** Counts of potentially pathogenic bacteria and fungi in the two containers after 1 month of fermentation, estimated by appropriate culture techniques.

**Bacteria and fungus**	**Container 1**	**Container 2**	**TAS 7023-2018 limit**
*Bacillus cereus*	< 10 CFU/g	< 10 CFU/g	1000 CFU/g
*Clostridium perfringens*	< 10 CFU/g	< 10 CFU/g	1000 CFU/g
*Escherichia coli*	< 3 MPN/g	< 3 MPN/g	10 MPN/g
*Salmonella* spp.	Not detected	Not detected	Not detected in 25 g
*Staphylococcus aureus*	< 10 CFU/g	< 10 CFU/g	1000 CFU/g
Yeast and molds	8.0 × 10^6^ CFU/g	9.0 × 10^7^ CFU/g	1000 CFU/g

*Note:* Microbiological criteria follow Thai Agricultural Standard TAS 7023‐2018 Pla‐Ra [28].

Abbreviations: CFU/g, colony‐forming units per gram; MPN/g, most probable number per gram.

### 3.2. Fermentation Alters the Composition and Diversity of Microorganisms in Pla‐Ra

To assess the impact of fermentation on *pla-ra′s* microbiome and mycobiome, we compared samples at 1 and 6 months after the start of fermentation, performing 16S rRNA and ITS sequencing after genomic DNA extraction (Figure 1a). Our findings showed that fermentation influenced alpha diversity in *pla-ra′s* microbiome. Bacterial alpha diversity significantly decreased after 6 months, with reductions in observed species richness (*p* = 0.0137) and the Shannon diversity index (*p* = 0.0016) (Figure 1b). This aligns with previous studies indicating a decline in bacterial diversity as fermentation progresses [30, 31]. Conversely, fungal alpha diversity increased, showing higher observed species richness (*p* = 0.0899) and a significant rise in the Shannon index (*p* = 0.024) (Figure 1c). This trend of decreasing bacterial but increasing fungal diversity suggests distinct environmental adaptations during fermentation. Beta diversity analysis using Bray–Curtis distances revealed significant differences between 1 and 6 months after fermentation groups for both bacterial (*p* = 0.048) and fungal communities (*p* = 0.006) (Figure 1c,d). The sharper shift in fungal communities indicates higher sensitivity to fermentation conditions, likely due to increased nutrient availability from bacterial metabolism. These findings point to a specialized bacterial community dominated by fermentative species and broader fungal diversity, suggesting a dynamic microbial succession during *pla-ra* fermentation, consistent with other fermented foods [32].

Figure 1Conceptual framework and microbial diversity analysis of *pla-ra* samples. (a) Conceptual framework of the study: an overview of the study design, indicating key stages from sample collection through microbial analysis. This figure was created by http://BioRender.com (license no. TU287PPRR6 in Data S1). (b) Alpha diversity (observed and Shannon index) of bacteria and fungi in *pla-*ra samples after 1 and 6 months of fermentation (*n* = 6 per time point). Linear regression models, adjusted for container effects, were applied to evaluate significance of alpha diversity indices. (c, d) Principal coordinate analyses (PCoA) of beta diversity in *pla-ra* samples at 1 and 6 months after start of fermentation (*n* = 6 per time point). The *p* values were calculated using PERMANOVA. Axes show the first two principal coordinates with the percent variance explained. Significance levels are indicated as  ^∗^ *p* < 0.05 and  ^∗∗^ *p* < 0.01.

**Figure:**
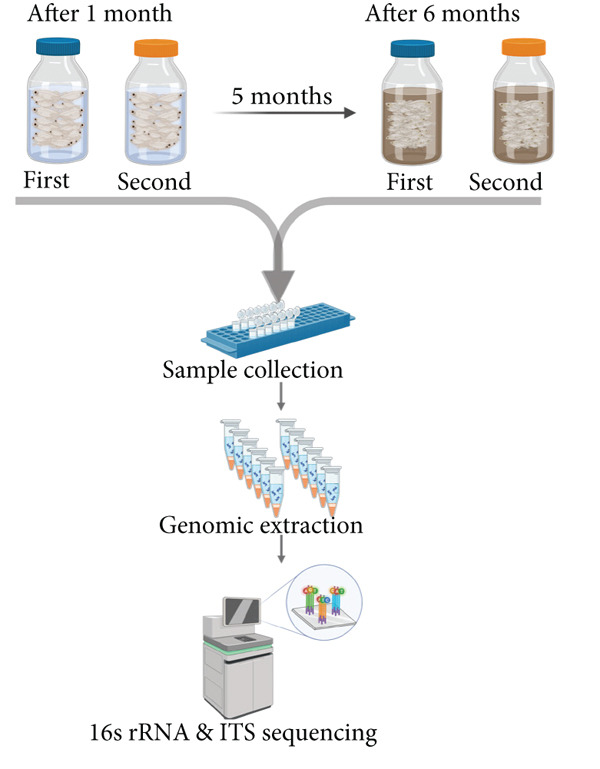
(a)

**Figure:**
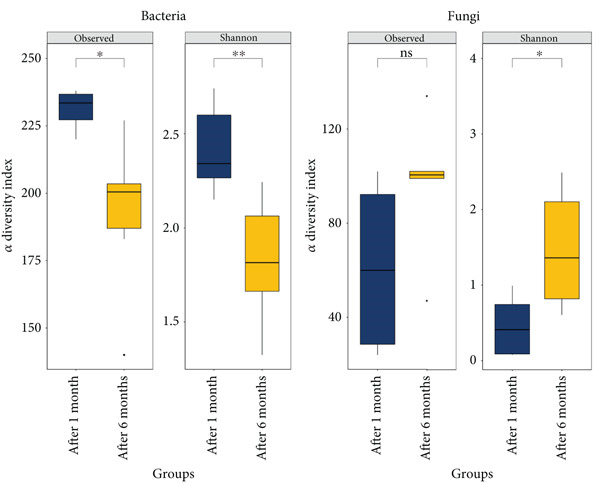
(b)

**Figure:**
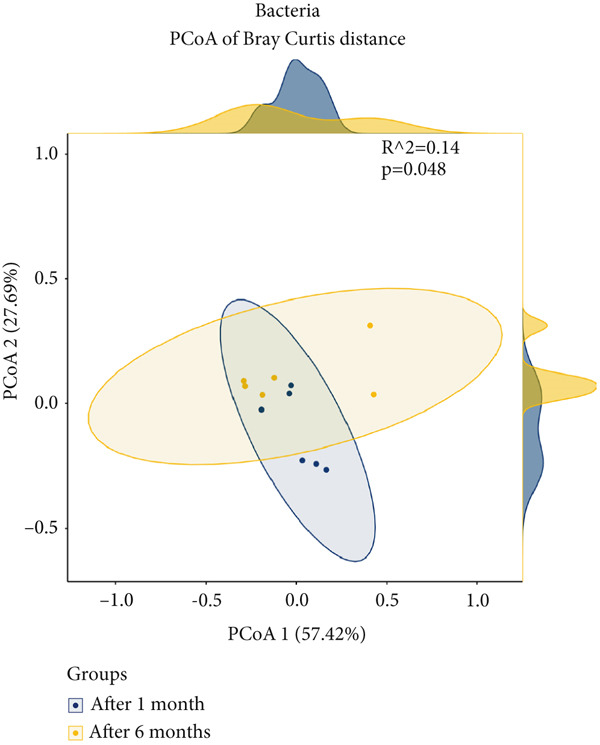
(c)

**Figure:**
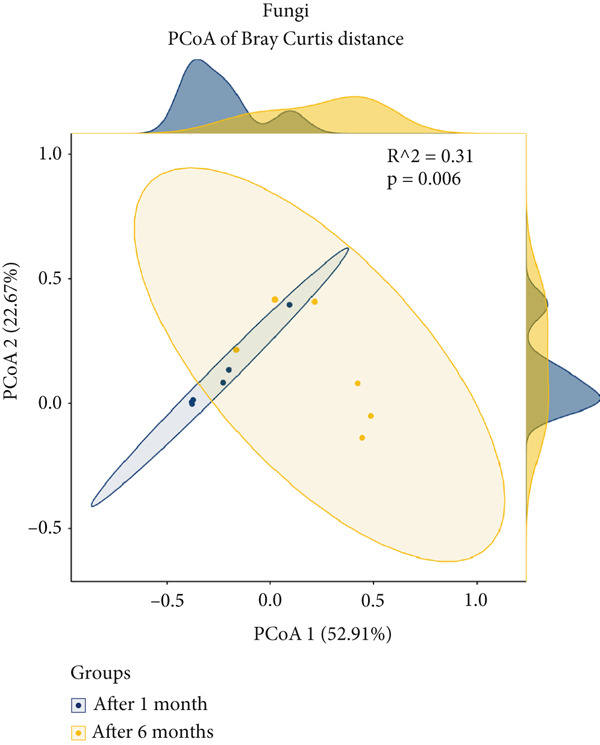
(d)

### 3.3. Influence of Fermentation on Composition of Bacterial and Fungal Communities in Pla‐Ra

Hereafter, significance refers to *p*adj < 0.1; taxa while values of *p*adj ≥ 0.1 are reported as trends only (full statistics in Data S2 Sheet 1). Differential abundance analysis revealed significant changes in the microbial community composition between *pla-ra* samples after 1 and 6 months of fermentation (Figure 2 and Data S2 Sheet 1). Six bacterial genera (Figure 2a) exhibited higher relative abundance after 1 month (*p* ≤ 0.05): *Candidatus Hydrogenedens* (*p* = 0.0014, *p*adj = 0.038), *Bellilinea* (*p* = 0.0043, *p*adj = 0.070), uncultured *Acidobacteria* Group 21 (*p* = 0.0055, *p*adj = 0.080), Group 22 (*p* = 0.0098, *p*adj = 0.099), Group 13 (*p* = 0.031, *p*adj = 0.238), and Group 17 (*p* = 0.043, *p*adj = 0.276). Conversely, nine genera showed higher relative abundance after 6 months of fermentation (*p* ≤ 0.05). These included *Halanaerobium* (*p* = 3.71e − 5, *p*adj = 0.0046), *Enhydrobacter* (*p* = 5.75e − 5, *p*adj = 0.0046), *Dermacoccus* (*p* = 0.0032, *p*adj = 0.063), *Virgibacillus* (*p* = 0.0067, *p*adj = 0.090), and *Brevibacterium* (*p* = 0.0076, *p*adj = 0.094). The *p* values of the remaining four genera in this category were at the “trend” level: *Brevundimonas* (*p* = 0.021, *p*adj = 0.192), *Rothia* (*p* = 0.033, *p*adj = 0.238), *Acetothermia_genera_incertae_sedis* (*p* = 0.046, *p*adj = 0.276), and *Staphylococcus* (*p* = 0.046, *p*adj = 0.276) (Data S2 Sheet 1). In the fungal community (Figure 2b), *Magnaporthe* trended toward higher relative abundance at 1 month (*p* = 0.014, *p*adj = 0.429), while *Penicillium* exhibited a trend toward increased relative abundance in fermented samples after 6 months (*p* = 0.0053, *p*adj = 0.275).

Figure 2Differential abundance of microbial genera in *pla-ra* after 1 and 6 months of fermentation. (a) Bacterial genera. (b) Fungal genera. Only genera with *p* < 0.05 are displayed. The *x*‐axis represents log2 fold change (log2FC) in abundance, with positive values indicating higher abundance in samples after 6 months and negative values indicating higher abundance after 1 month of fermentation. The *y*‐axis lists the differentially abundant genera. Significance levels are represented as  ^∗^ *p*adj < 0.1. Abbreviations: log2FC = log2 fold change; *p*adj = adjusted *p* value (Benjamini–Hochberg). Sample size: *n* = 6 per time point.

**Figure:**
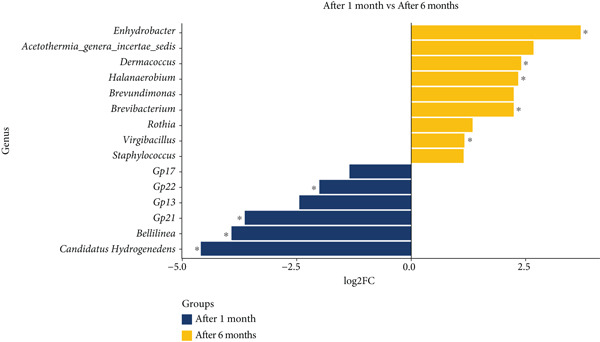
(a)

**Figure:**
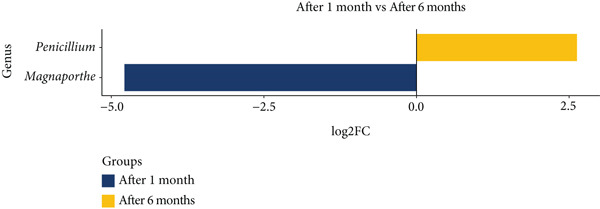
(b)

The higher abundance of *Candidatus Hydrogenedens*, *Bellilinea*, and *Acidobacteria* group taxa (lactic acid subgroups of *Acidobacteria*) [33, 34] in the lower‐pH conditions 1 month after the start of fermentation suggests their role in acid production during the initial phase of *pla-ra* fermentation. Although *Acidobacteria* Groups 13 and 17 did not meet the multiple‐testing threshold, their transient enrichment at 1 month is biologically plausible given that *Acidobacteria* include acid‐tolerant, oligotrophic heterotrophs capable of persisting in low‐pH, early‐fermentation niches [35–37]. Their decline by 6 months may reflect a reduced tolerance to changing environmental conditions, possibly influenced by slightly increased salt concentrations and decreased oxygen availability [32]. In this context, *Enhydrobacter* contributes to flavor development through carbohydrate and amino acid metabolism. While its specific role in fish fermentation is not fully elucidated, its increased abundance in fermented *pla-ra* suggests potential involvement in flavor formation. The presence of *Enhydrobacter* in both vinegar and fermented fish highlights its versatility in various fermented food products [14, 38]. Further research using metagenomic and metabolomic approaches could clarify the specific contribution of *Enhydrobacter* to the sensory characteristics of *pla-ra. Dermacoccus* and *Virgibacillus* may also play a role in flavor development and textural changes during fermentation [39], although their specific contributions to *pla-ra* characteristics require further investigation. The significant increase in halophilic bacteria, particularly *Halanaerobium*, with increased salt content after 6 months of fermentation, is consistent with adaptation to the high‐salt environment typical of fermented fish products [40]. *Halanaerobium* species are known for their ability to grow in hypersaline conditions and may contribute to the characteristic flavors of fermented fish products through their metabolic activities, including the production of organic acids and volatile compounds [41]. The increased representation of *Brevibacterium*, known for its proteolytic activities, may contribute to protein degradation during fermentation, potentially influencing the product′s taste and smell [42]. *Brevibacterium* species have been associated with the production of volatile sulfur compounds, which can contribute to the distinct aroma profile of fermented foods [43]. The presence of *Staphylococcus* at both 1 and 6 months, with a slight increase in samples after 6 months, is consistent with findings in other fermented fish products. *Staphylococcus* species, particularly coagulase‐negative staphylococci (CNS), are common in traditional fermented foods and can contribute positively to product characteristics [44]. In fermented fish products, *Staphylococcus* has been associated with the production of flavor compounds and the inhibition of biogenic amine formation [45]. Additionally, some *Staphylococcus* strains isolated from fermented foods have shown probiotic potential and antimicrobial activities against foodborne pathogens [46]. The presence of *Staphylococcus* in *pla-ra* may contribute to the product′s unique sensory profile and potentially its preservation. CNS are well documented and can influence aroma via proteolysis/lipolysis and, in some matrices, reduce biogenic amines [47–49]. The shift toward halophilic and proteolytic bacteria suggests a fermentation process that favors salt‐tolerant species capable of breaking down proteins, which is consistent with the traditional preparation methods of *pla-ra*.

In the fungal community, the shift from dominance by *Magnaporthe* in samples after 1 month to *Penicillium* at the end of 6 months highlights the changing ecological dynamics during fermentation. *Magnaporthe* species are typically associated with plant pathogenesis. Their presence in *pla-ra* samples at 1 month suggests they may have been introduced with raw materials.

The observation that fungal loads at 1 month (8.0 × 10^6^–9.0 × 10^7^ CFU/g) substantially exceeded the common regulatory guideline of 1000 CFU/g [28] raises a significant food safety consideration regarding traditional *pla-ra* production. This consideration is further underscored by the microbial succession to a *Penicillium*‐dominant mycobiome after 6 months of fermentation. While certain *Penicillium* species are integral to maturation and flavor development in some fermented foods, the genus also includes well‐documented mycotoxigenic species, posing a potential health risk to consumers [50]. A limitation of the present study is that ITS2 amplicon sequencing, while effective for genus‐level identification, often lacks the resolution to distinguish closely related *Penicillium* species. Robust species‐level discrimination in future work should employ full‐length ITS long‐read amplicons and/or species‐specific PCR, enabling assessment of both sensory and safety implications [51, 52]. To enhance the safety and consistency of the final product, mitigation strategies could be implemented. These may include the application of selected starter cultures to enhance product safety by inhibiting the growth of undesirable microorganisms [53, 54]. For safety reasons, we recommend future investigations incorporate direct chemical analysis for prevalent mycotoxins (e.g., aflatoxins and ochratoxin A) [55]. In addition, process‐based safety controls should be considered, including maintaining adequate salt concentration, low amount of free water availability for microbes′ use, and appropriate pH and temperature conditions, together with hygienic container handling. These strategies provide a more comprehensive framework for managing both mycotoxins and undesirable microbes in traditional *pla-ra* fermentation.

### 3.4. Microbial Network Restructuring During Pla‐Ra Fermentation

Differential gene correlation analysis (DGCA) revealed significant restructuring of microbial networks during *pla-ra* fermentation. Network analysis identified distinct modules after 1 and 6 months of fermentation: three involving bacteria–bacteria interactions (Figure 3a), two showing interactions between fungi (Figure 3b), and four involving bacteria–fungi networks (Figure 3c). In the bacteria–bacteria network, genera that significantly differed in abundance between 1 and 6 months are shown as red nodes. In the fungi–fungi network, major fungal genera are represented in blue. In the bacteria–fungi network, significantly different bacterial genera are indicated in pink, while significant fungal genera are displayed in purple. Genera without significant differences are shown as gray nodes.

Figure 3Differential correlation analysis of microbial genera in *pla-ra* samples after 1 and 6 months of fermentation. (a) Bacteria–bacteria interactions. (b) Fungi–fungi interactions. (c) Bacteria–fungi interactions. Edge colors represent the direction of correlation after 1 and 6 months of fermentation, with the count indicating the number of pairs of genera exhibiting each correlation pattern. Only pairs of genera with significant differential correlations (*p* ≤ 0.05, permutation test) are displayed. Node background colors denote significant differential abundance between 1 and 6 months after start of fermentation (*p* ≤ 0.05, DESeq2): red for bacterial genera, blue for fungal genera, pink for bacterial genera in the bacteria–fungi network, and purple for fungal genera in the bacteria–fungi network. Interactions are classified as follows: no correlation after 1 month to positive after 6 months (0/+); no correlation to negative (0/−); negative to no correlation (−/0); positive to no correlation (+/0); negative to positive (−/+); and positive to negative (+/−). Sample size: *n* = 6 per time point.

**Figure:**
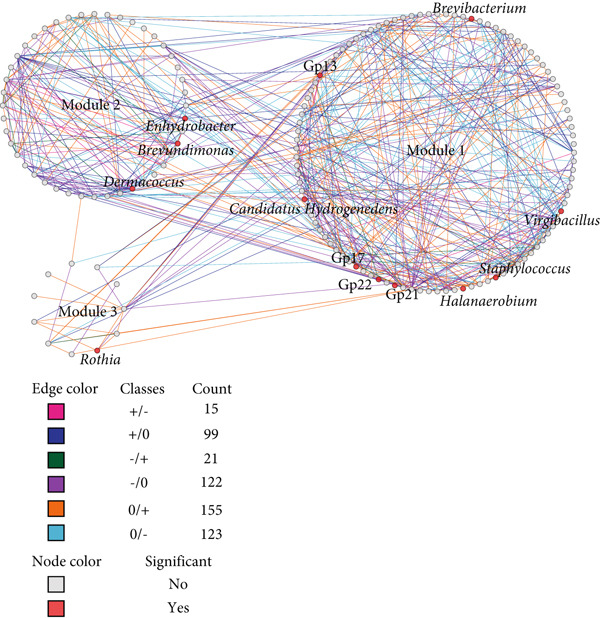
(a)

**Figure:**
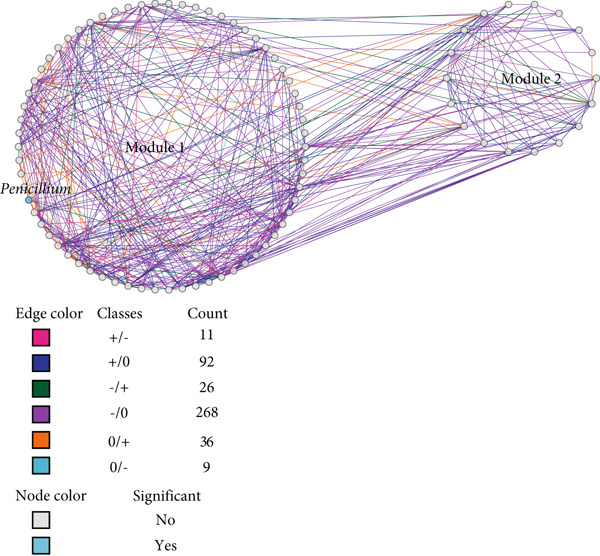
(b)

**Figure:**
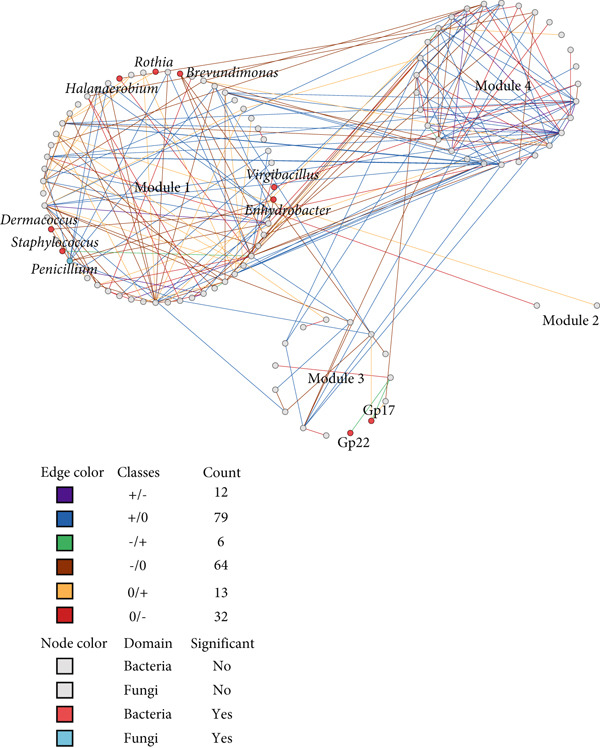
(c)

In these networks, six sign‐pair classes were identified for pairwise correlations in relative abundance when comparing samples at 1 and 6 months after fermentation. The +/− class includes cases where two taxa were positively correlated in abundance at 1 month but negatively correlated at 6 months; +/0 denotes a positive correlation at 1 month that disappeared by 6 months; −/+ denotes the opposite—negative at 1 month and positive at 6 months; −/0 represents a negative correlation at 1 month that was not apparent at 6 months; 0/+ indicates no correlation at 1 month but a positive correlation at 6 months; and 0/− indicates no correlation at 1 month but a negative correlation at 6 months. For illustration, 0/+ pairs (such as *Rothia–Nesterenkonia*, Figure 3a and Data S2 Sheet 4) suggest late‐emerging facilitation/cross‐feeding, −/0 pairs (such as *Penicillium*–*Didymella*, Figure 3b and Data S2 Sheet 5) indicate transient early inhibition, and +/− pairs (such as *Acidobacteria* Group 13–*Micrococcus*, Figure 3a and Data S2 Sheet 6) reflect a later shift toward competition or niche replacement.

The first network (Figure 3a) contained 14 genera, with significant differential abundance (DESeq2, *p* ≤ 0.05) between 1 and 6 months after the start of fermentation. In the bacteria–bacteria network, several key bacterial genera were identified as nodes. *Candidatus Hydrogenedens* had a +/0 relationship with *Corynebacterium* (Data S2 Sheet 4). Both types of bacteria can change nitrate into ammonia [56, 57]. These findings suggest that *Candidatus Hydrogenedens* and *Corynebacterium* may help lower nitrates in the initial stage of *pla-ra* fermentation. Similarly, *Gemella* and *Acidobacteria* Group 13 also exhibited a +/0 relationship. *Gemella* species can ferment glucose into lactate and acetate as major metabolic end products [58]. Additionally, Group 13 can produce lactic acid [59], which helps keep microbes stable and inhibit the growth of toxins in food. Our results suggest that *Gemella* and Group 13 play an initial role in lactic acid production during *pla-ra* fermentation.

Interestingly, in the most prevalent 0/+ correlation pattern, the key bacteria included *Rothia*, *Candidatus Hydrogenedens*, *Staphylococcus*, and *Brevibacterium*. *Rothia* exhibited a 0/+ interaction with *Nesterenkonia* and *Kocuria*, with both *Rothia* and *Nesterenkonia* able to metabolize glucose into acids, including acetic and butyric acids [60, 61]. This metabolic activity likely contributes to the aromatic flavor and acidic taste of *pla-ra*. *Kocuria*, similar to *Nesterenkonia*, influences the sugar content and acidity in fermented products such as saké [62], enhancing the sour taste of *pla-ra* by supporting *Rothia* during fermentation. *Staphylococcus* exhibited a 0/+ interaction with *Escherichia/Shigella* and *Flintibacter*. These last two taxa contribute to the fermentation of various fermented foods by enhancing acidity, ester aroma, and alcohol aroma [63, 64]. Our results confirm that *Staphylococcus* and *Escherichia/Shigella* may also play a role in enhancing acidity and aroma in *pla-ra*. This enhancement is likely due to their ability to metabolize carbohydrates and amino acids into organic acids, esters, and volatile compounds, which influence the final sensory characteristics of the product [32, 63, 65, 66].

*Enhydrobacter* exhibited a 0/− interaction with *Halomonas*, suggesting a potential competitive or inhibitory relationship between these genera. *Enhydrobacter* has been identified in various fermentation processes and is known for its role in metabolic transformations, particularly in carbohydrate and protein metabolism [67, 68]. *Halomonas* species, being halophilic bacteria, are commonly associated with high‐salt and high‐pH fermented foods and contribute to the production of osmolytes and other fermentation‐related compounds [69, 70]. However, in environments where *Enhydrobacter* thrives, *Halomonas* may be lower in abundance. Another 0/− interaction was found between *Halanaerobium* and *Blautia*. *Halanaerobium* is extensively documented in high‐salt and anaerobic fermentation environments [71], where it is a significant player in protein metabolism, producing volatile fatty acids (VFAs) such as acetate, propionate, and butyrate, as well as hydrogen sulfide (H_2_S) and ammonia [72]. In contrast, *Blautia* is primarily associated with carbohydrate fermentation, where it contributes to the stability of the gut and food microbiota by producing acetate as a primary metabolite [73, 74]. *Halanaerobium* may suppress *Blautia* through inhibitory metabolites like H_2_S and alterations in pH and nutrient availability. This shift could influence acidity, volatile compound production, and overall fermentation characteristics.

Some key bacteria exhibited a +/− relationship with each other, including Group 13 with *Micrococcus* and Group 22 with *Escherichia/Shigella*. *Micrococcus* and *Escherichia/Shigella* have the potential for production of acid under anaerobic conditions [75–77]. The higher acidity at 1 month after fermentation may promote the growth of *Acidobacteria* Group 13 and lactic acid bacteria Group 22 [34]. Nonetheless, this association transitioned to a negative correlation after 6 months of fermentation, suggesting that competition for nutrients, alterations in metabolic activity, or environmental changes may have resulted in the suppression of Group 13 and Group 22 in favor of other dominating microbial populations.

*Penicillium* had many connections in our network of fungal–fungal interactions (Figure 3b), while *Cystodendron* and *Didymella* were the only genera that were identified. *Penicillium* was negatively correlated with these fungi during the first stage of fermentation, as shown by the −/0 correlation. However, no interaction was seen after 6 months of fermentation most likely because of microbial succession, substrate depletion, or changes in the environment. *Penicillium* has long been used to help ferment foods by improving their nutritional value, flavor, and microbial stability [78, 79]. *Penicillium* may outcompete other fungi, such as *Cystodendron* and *Didymella*, by producing organic acids or antimicrobial compounds [80]. This likely contributes to its high abundance in fermented *pla-ra*.

This is the first study to report correlations between bacteria and fungi in *pla-ra* (Figure 3c). The bacterial–fungal network analysis revealed key interactions, including *Penicillium* with *Dermacoccus* and *Enhydrobacter*, exhibiting a −/0 relationship. This type of relationship indicates that *Penicillium* initially inhibited the growth of these bacteria, but the effect diminished as fermentation progressed. *Penicillium* species are known to produce organic acids, such as citric and gluconic acids, which can create localized pH gradients that inhibit bacterial growth [80, 81]. The dynamic alteration from a negative correlation to no interaction after 6 months of fermentation is likely due to microbial succession, a common process in fermented foods where early microorganisms modify the environment, altering the microbial composition and creating conditions that favor succeeding microbial populations [82].

### 3.5. KEGG Pathways and Correlation With Bacterial Genera Significantly Altered in Abundance During Fermentation of Pla‐Ra

As shown in Figure 4a and Data S2 Sheet 3, 11 KEGG pathways were significantly different (DESeq2, *p* < 0.05) in *pla-*ra samples between 1 and 6 months after start of fermentation. Seven KEGG pathways were notably more active after 6 months. These included glycerophospholipid metabolism, pentose and glucuronate interconversions, ascorbate and aldarate metabolism, restriction enzyme, phosphatidylinositol signaling system, tropane, piperidine and pyridine alkaloid biosynthesis, and steroid biosynthesis. Glycerophospholipid metabolism is a key factor in improving the flavor of dry‐fermented tilapia sausages [83]. The upregulation of this pathway at 6 months after start of fermentation suggests that the fermentation process is still continuing. Strong ascorbate and aldarate metabolism at 6 months further implies that this pathway plays a significant role in flavor formation, aligning with a previous report [84]. The observed increase in tropane, piperidine, and pyridine alkaloid biosynthesis is consistent with previous findings in kinema, a naturally fermented soybean food from the Eastern Himalayas [85]. The ability of several bacteria to synthesize steroids has been well documented [86], with some steroids exhibiting antipathogenic properties [87]. The significant increase in steroid biosynthesis in fermented *pla-ra* at 6 months suggests that some bacteria may produce these compounds to inhibit pathogenic bacteria during fermentation [88].

Figure 4Comparisons of metabolic pathway activity in *pla-ra* between 1 and 6 months of fermentation, along with a correlation analysis between bacterial genera and KEGG pathways. (a) Comparison of KEGG pathway abundances between 1 and 6 months. The *x*‐axis is ordered by time of fermentation. Note that six samples were analyzed at each timepoint. The heatmap displays *z*‐score normalized KEGG pathway abundances, with high abundance shown in brown and low abundance in dark green. (b) Heatmap showing Spearman correlations between significantly different bacterial genera (*x*‐axis) and significantly different KEGG pathways (*y*‐axis). Correlation values are represented by different colors in the graph. The color scale on the right shows the color partitioning of the different *R* values, ranging from −0.5 (navy, negative correlation) to 0.5 (yellow, positive correlation). Abbreviations: log2FC = log2 fold change; *p*adj = adjusted *p* value. Significance coding: ∗ indicates *p* < 0.05 with adjusted *p*adj > 0.1.

**Figure:**
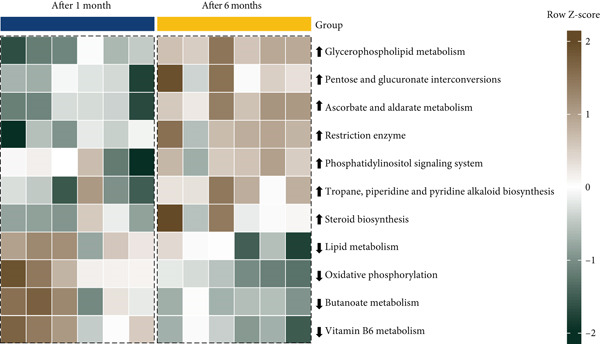
(a)

**Figure:**
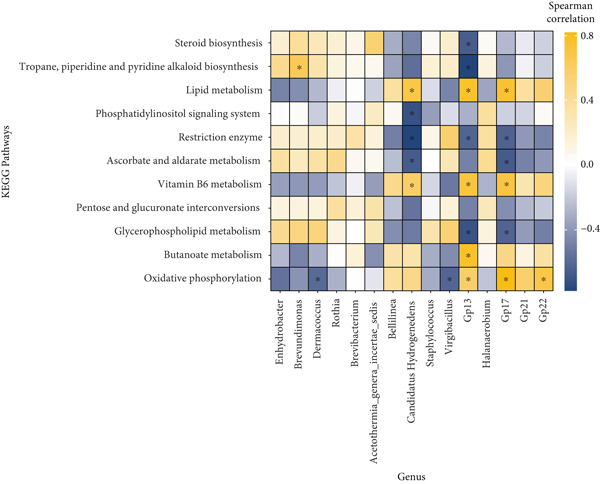
(b)

*Pla-ra* fermentation significantly reduced the representation of several KEGG pathways over time, including lipid metabolism, oxidative phosphorylation, butanoate metabolism, and vitamin B6 metabolism. Lipid metabolism is a common feature of fermentation processes in various fermented foods, such as certain sausages, where it influences the flavor and quality of the final product [89]. The higher levels of lipid and butanoate metabolism after 1 month of fermentation suggest the rapid initiation of lipid‐derived and short‐chain fatty acid (SCFA) production. Specifically, the elevated butanoate (butyrate) metabolism, known to be associated with SCFA production [90], indicates that SCFAs may begin forming early in the fermentation process. In addition, the observed decrease in vitamin B6 metabolism after 6 months aligns with previous studies reporting reduced B vitamins, including vitamin B6, in fermented foods such as kefir, a type of fermented milk [91].

Spearman correlation analysis of significantly altered bacterial genera in *pla-ra* (Figure 4b and Data S2 Sheet 7) revealed both positive correlations (yellow), indicating genera that were positively associated with specific pathways, and negative correlations (navy), representing negative relationships (*p* < 0.05). *Brevundimonas* positively correlated with tropane, piperidine, and pyridine alkaloid biosynthesis, suggesting its possible role in producing these bioactive compounds. This aligns with previous research on microbial alkaloid metabolism in fermented foods like kinema, based on soybeans [85]. Additionally, *Enhydrobacter*, *Brevundimonas*, and *Dermacoccus* positively correlated with glycerophospholipid metabolism, indicating their role in flavor development in *pla-ra*, similar to dry‐fermented tilapia sausage*s* [83]. Furthermore, *Candidatus Hydrogenedens*, Group 13, and Group 17 were positively associated with lipid metabolism, suggesting their involvement in lipid‐derived flavor formation at 1 month after the start of *pla-ra* fermentation, consistent with findings in other fermented foods [89]. The positive correlation of *Enhydrobacter*, *Brevundimonas*, *Dermacoccus*, and *Rothia* with ascorbate and aldarate metabolism suggests their possible role in flavor development during *pla-ra* fermentation. Ascorbate and aldarate metabolism contributes to the production of organic acids and volatile compounds that enhance the sensory characteristics of fermented foods, in line with previous findings that microbial activity in fermented fish products influences flavor compound formation through this metabolic pathway [84, 92].

Because PICRUSt2 uses 16S rRNA profiles to predict functions, our KEGG results are inferential only. How reliable these predictions are depends on the number and closeness of available reference genomes (summarized by the NSTI): reliability drops for lineages with few or distant references. The method also assumes similar gene content within a taxon, which can vary because of strain differences and horizontal gene transfer. In addition, it does not measure gene expression or enzyme activity, so differences indicate potential capacity only. Finally, the pipeline focuses on bacteria and does not include fungal pathways, even though fungi are important in *pla-ra*. We therefore report these as differences in predicted potential and interpret them with caution. To verify these patterns, future work should pair the predictions with metabolomics (LC–MS/GC–MS of organic acids, amino acid derivatives, biogenic amines, and key volatiles) and metatranscriptomics or targeted RT‐qPCR. Where possible, shotgun metagenomics could also test gene content differences at the strain level.

## 4. Conclusions

This study is the first to examine bacterial–fungal interactions during *pla-ra* fermentation. Length of fermentation influenced microbial diversity, cross‐kingdom network structure, and predicted metabolic pathways. These shifts likely contribute to sensory attributes of *pla-ra*. Key microbial genera and candidate pathways were identified that can guide the refinement of traditional practice. Future work should isolate and functionally test key taxa, particularly major microbial taxa in high‐quality *pla-ra* in controlled inoculation trials. Microbial community profiling should be paired with untargeted and targeted metabolomics or volatilomics (LC–MS/GC–MS) to validate pathway predictions and identify marker compounds. Sensory evaluation by a trained panel should link taxa and metabolites to flavor and acceptance. Where appropriate, shotgun metagenomics and, if needed, metatranscriptomics should provide strain‐level resolution and pathway reconstruction. Safety‐focused measurements such as mycotoxin quantification and evaluation of process variables and starter‐culture strategies should support consistent and safe production.

## Disclosure

This article has been prepared with complete academic and professional independence. The final manuscript was reviewed and approved by all authors. All authors agree to be accountable for all aspects of work ensuring integrity and accuracy.

## Conflicts of Interest

The authors declare no conflicts of interest.

## Author Contributions

Thatsanapong Pongking: conceptualization, data curation, formal analysis, methodology, roles/writing—original draft. Xiuqiang Chen and Keerapach Tunbenjasiri: conceptualization, investigation, validation, writing—review and editing. Lu Zhang: investigation, validation, methodology and resources. Ratthaphol Kraiklang, Somchai Pinlaor, Arunnee Sangka, Aroonlug Lulitanond, and David Blair: resources, investigation, methodology. Porntip Pinlaor: funding acquisition, project administration, resources, supervision, validation, visualization, writing—review and editing.

## Funding

This research was supported by Khon Kaen University Research Fund (I62‐00‐24‐05) and the NSRF via the Program Management Unit for Human Resources and Institutional Development and the Reinventing University 2025 through Khon Kaen University, Thailand. Thatsanapong Pongking and Porntip Pinlaor were supported by the National Research Council of Thailand (NRCT) through NRCT5‐RGJ63003‐055.

## Supporting information


**Supporting Information** Additional supporting information can be found online in the Supporting Information section. The supporting information includes Data S1 (BioRender licensing documentation). Data S2 (Excel) contains a front‐page README and contents checklist, a full sample metadata sheet, and nine data sheets: full DESeq2 outputs for bacterial taxa (Sheet 1), fungal taxa (Sheet 2), and KEGG Level 3 pathways (Sheet 3); DGCA results for bacteria–bacteria (Sheet 4), fungi–fungi (Sheet 5), and bacteria–fungi (Sheet 6); a Spearman correlation matrix linking significant bacteria to KEGG pathways (Sheet 7); and OTU count matrices for 16S (Sheet 8) and ITS2 (Sheet 9).

## Data Availability

The raw amplicon sequencing data has been deposited in NCBI as BioProject PRJNA1253690.
